# The Expansion Segments of 28S Ribosomal RNA Extensively Match Human Messenger RNAs

**DOI:** 10.3389/fgene.2018.00066

**Published:** 2018-03-07

**Authors:** Michael S. Parker, Ambikaipakan Balasubramaniam, Floyd R. Sallee, Steven L. Parker

**Affiliations:** ^1^Department of Microbiology and Molecular Cell Sciences, University of Memphis, Memphis, TN, United States; ^2^Department of Surgery, University of Cincinnati School of Medicine, Cincinnati, OH, United States; ^3^Department of Psychiatry, University of Cincinnati School of Medicine, Cincinnati, OH, United States; ^4^Department of Pharmacology, University of Tennessee Health Science Center, Memphis, TN, United States

**Keywords:** GC content, rRNA/mRNA matches, RNA expansion segment, RNA nucleotide bias, RNA nucleotide repeat

## Abstract

Eukaryote ribosomal RNAs (rRNAs) have expanded in the course of phylogeny by addition of nucleotides in specific insertion areas, the expansion segments. These number about 40 in the larger (25–28S) rRNA (up to 2,400 nucleotides), and about 12 in the smaller (18S) rRNA (<700 nucleotides). Expansion of the larger rRNA shows a clear phylogenetic increase, with a dramatic rise in mammals and especially in hominids. Substantial portions of expansion segments in this RNA are not bound to ribosomal proteins, and may engage extraneous interactants, including messenger RNAs (mRNAs). Studies on the ribosome-mRNA interaction have focused on proteins of the smaller ribosomal subunit, with some examination of 18S rRNA. However, the expansion segments of human 28S rRNA show much higher density and numbers of mRNA matches than those of 18S rRNA, and also a higher density and match numbers than its own core parts. We have studied that with frequent and potentially stable matches containing 7–15 nucleotides. The expansion segments of 28S rRNA average more than 50 matches per mRNA even assuming only 5% of their sequence as available for such interaction. Large expansion segments 7, 15, and 27 of 28S rRNA also have copious long (≥10-nucleotide) matches to most human mRNAs, with frequencies much higher than in other 28S rRNA parts. Expansion segments 7 and 27 and especially segment 15 of 28S rRNA show large size increase in mammals compared to other metazoans, which could reflect a gain of function related to interaction with non-ribosomal partners. The 28S rRNA expansion segment 15 shows very high increments in size, guanosine, and cytidine nucleotide content and mRNA matching in mammals, and especially in hominids. With these segments (but not with other 28S rRNA or any 18S rRNA expansion segments) the density and number of matches are much higher in 5′-terminal than in 3′-terminal untranslated mRNA regions, which may relate to mRNA mobilization via 5′ termini. Matches in the expansion segments 7, 15, and 27 of human 28S rRNA appear as candidates for general interaction with mRNAs, especially those associated with intracellular matrices such as the endoplasmic reticulum.

## Introduction

Traditionally, interaction of mRNAs with ribosomes is assumed to involve proteins of the smaller subunit (SSU), using short mRNA tracts (the “internal ribosome entry sites,” IRES) for an initial positioning. No generalized involvement of either the 18S rRNA or of RNAs of the larger subunit (LSU) has been established thus far. However, some elements of 18S rRNA could be contacting mRNA in the vicinity of the ribosomal entry site (Pisarev et al., 2008; Pánek et al., 2013). The large rRNA of mammalian LSU (28S rRNA) was shown to hybridize with 5.8S and 5S rRNAs and with polyA (+) RNAs (Maxwell and Martin, 1986) and to have several complementary 5′utr motifs with ferritin mRNA (Jain et al., 1985). The 5.8S ribosomal RNA is extensively associated with 28S rRNA (Noller et al., 1981; for a detailed model see the supplement of Chandramouli et al., 2008), which constitutes a ubiquitous example of a massive and tight canonical interaction of the large LSU rRNA with a different RNA molecule. The large LSU RNA should also have direct dynamic canonical contacts with tRNAs (Meskauskas and Dinman, 2008). Most of the core and parts of expansion segments in rRNAs are associated with ribosomal proteins and therefore are not viewed as mRNA targets. However, it should be emphasized that considerable portions of LSU large expansion segments are not stably masked by proteins (Wakeman and Maden, 1989; Larsson and Nygård, 2001; Nygård et al., 2006; Chandramouli et al., 2008; Armache et al., 2010; Klinge et al., 2011) or by known RNA counterparts within the 60S subunit. The potential matching of unstructured mRNAs by short tracts of other RNAs generally estimates to a large frequency (see e.g., Parker et al., 2016 for possible 7–15 nt matches with microRNAs) and similar could be expected for rRNAs.

Compared to prokaryote 23S rRNAs, the large LSU rRNAs in eukaryotes show enlargement of up to 2,400 nucleotides (or up to 80% additional sequence), with a remarkable phylogenetically linked increase in both size and nucleotide bias from yeast to man (Chandramouli et al., 2008; Ben-Shem et al., 2010; Parker et al., 2015). This increase is linked to expansion segments added at strongly conserved insertion points, which enables use of the most expanded LSU RNA, human 28S rRNA, as a template for marking the expansion segment boundaries in 25-28S rRNAs across eukarya (Parker et al., 2015).

Evolution of eukaryotic rRNAs proceeded via insertions in prokaryote-related core sequences and further enlargement of the inserts (Noller et al., 1981; Stiegler et al., 1981; Gupta et al., 1983; Clark et al., 1984; Hassouna et al., 1984; Michot et al., 1984; Wakeman and Maden, 1989; Gerbi, 1996; Chandramouli et al., 2008; Armache et al., 2010; Ben-Shem et al., 2011; Klinge et al., 2011). Some of the expansion segments (ES) of both 18S rRNA (here abbreviated ESS) and 25S rRNA (ESL) of lower eukaryotes were shown to be essential for normal cell growth, and even for cell survival, in multiple contexts (Sweeney et al., 1994; Jeeninga et al., 1997; Van Nues et al., 1997; Ramesh and Woolford, 2016). Indispensability of either the ESS or the much larger ESL as yet needs a demonstration in metazoan cells. A GC- or AU-biased expansion of rRNAs has developed in both plants and metazoa, with GC bias largely being preferred. In the 25–28S RNAs of the large ribosomal subunit (LSU) several ESL are large even in lower eukarya. Large ESL are found in the tetrapod vertebrates, and very large ESL have developed in hominids, with a substantial enlargement even between the hominid apes and man (as will be indicated in this paper). In human 28S rRNA there are eight ESL of more than 50 nucleotides (nt), and two ESL of more than 700 nt, compared with four >50-nt expansion segments in 18S rRNA (abbreviated ESS; none larger than 180 nt) (Wakeman and Maden, 1989; Chandramouli et al., 2008). The large ESL are substantially exposed at the ribosome surface (Wakeman and Maden, 1989; Larsson and Nygård, 2001; Nygård et al., 2006; Chandramouli et al., 2008; Armache et al., 2010; Klinge et al., 2011), feature tracts that are not stably associated with ribosomal proteins (Larsson and Nygård, 2001; Chandramouli et al., 2008), and could be available for association with mRNAs as well as with non-ribosomal proteins and intracellular matrices (Parker et al., 2014, 2015). The ESS appear to have no firm general pattern and could differ much in subdivisions across species (Chandramouli et al., 2008; Ben-Shem et al., 2011; Parker et al., 2015; Quade et al., 2015).

Most of the expanded LSU rRNA sequence and of new ribosomal protein material in yeast are located on ribosome surface, encasing the evolutionarily conserved core (Ben-Shem et al., 2011). A very similar situation seems to obtain with three major ES of mammalian 28S rRNA, ESL7, ESL15, and ESL27 (Nygård et al., 2006; Chandramouli et al., 2008; Armache et al., 2010). Parts of these segments are highly mobile and are not clearly associated with ribosomal proteins in crystals of single ribosomes (Chandramouli et al., 2008; Armache et al., 2010). These parts conceivably could also be preferentially available for interaction with non-ribosomal proteins and RNAs.

Expansion of the large LSU RNA could be linked to association of the large subunit with intracellular matrices. In mammalian tissues such as liver, a major fraction of LSU is firmly attached to the endoplasmic reticulum (ER) membranes (Sabatini et al., 1966), without critical participation of mRNA (Kruppa and Sabatini, 1977). An interaction of the 60S subunit with mRNA is only rarely assumed (Sloma and Nygård, 2001) although ESL are known to interact with extraribosomal entities (Leidig et al., 2013). The extremely GC-rich ES of vertebrate 28S rRNA (Clark et al., 1984; Wakeman and Maden, 1989; Chandramouli et al., 2008), which also are rich in G and C repeats (homoiterons; see Parker et al., 2015), may have roles in mobilization of mRNAs from mRNPs, by analogy e.g., with mRNP protein binding by polyriboguanylate (Barrieux and Rosenfeld, 1977); rRNA guanine is important in codon association with either 16S rRNA of prokaryotes or 18S rRNA of eukaryotes (Demeshkina et al., 2000) and G-rich rRNA motifs could generally complement mRNAs (Barendt et al., 2013). The mRNA sequences used in initial positioning on the ribosome (IRES sites) could interact with RNAs of either subunit. The paucity of precisely defined IRES in mammalian non-viral mRNAs may relate to a generalized supportive involvement of the considerable portions of ESL that are exposed on the LSU surface (see Larsson and Nygård, 2001; Chandramouli et al., 2008) for description of these LSU parts). Tracts of ESL also might be able to recognize and position mRNAs from mRNPs associated with intracellular matrices, including the ER as well as the cytoskeleton (Bassell et al., 1994; Vedeler and Hollås, 2000; Villacé et al., 2004). As will be shown in this study, compared to other human rRNA segments the ESL have much larger capacity for interaction with mRNAs either in terms of total matches, or with respect to match density.

## Methods

### The RNA sequences examined

Ribosomal RNA sequences were retrieved from Entrez nucleotide database (https://www.ncbi.nlm.nih.gov/nuccore), with the aid of access codes from Comparative RNA Web site (CRW; http://www.rna.icmb.utexas.edu). The rRNA sequences examined are listed in the Table S1. Human mRNA sequences were retrieved from the Ensembl database (http://www.ensembl.org). A total of 18,810 mRNAs with matching 5′utr, cds and 3′utr sectors (from 17,392 named protein-coding genes) from 2015 lists in Ensembl database were examined. Average numbers of nucleotides in the examined mRNA sectors are 250 for 5′utr, 1,678 for cds and 1,474 for 3′utr. An additional examination of human protein-coding mRNAs available as of 8/2017 (see Table S3) indicated that the 2015 collection used in this study is sufficiently representative of the potential of matching to ribosomal RNAs.

### Boundaries of the expansion segments

This study utilized the numbering of rRNA expansion segments described by Gerbi (1996) and Yokoyama and Suzuki (2008). The segment boundaries were derived from Chandramouli et al. (2008) and Wakeman and Maden (1989) (see Parker et al., 2015) and are listed in Table S2. The segment boundaries of human rRNAs were searched for in *clustalW* (http://www.expasy.org) alignments with other ribosomal RNAs to score the matching starting and ending nucleotides. This approach defined segments that for the studied 25-28S rRNAs (see Table S1 and **Table 3**) correspond well with published values from modeling of RNA structure. It should be noted that the ESS boundaries are preliminary, which relates to the structural diversity of eukaryote 18S rRNAs (Xie et al., 2011). The core segments are numbered in tandem with the expansion segments that follow in rRNA sequences; thus, CSL5 precedes ESL5 and CSL41 precedes ESL41. The same relative labeling was used for 18S core segments (CSS). The sequence-ending CSLend and CSSend follow, respectively, ESL41 and ESS12. Expansion segments of 28S rRNA that have less than six nucleotides were included in the surrounding core segments. The expansion segments ES1–ES3 in the 5.8S rRNA/28S rRNA complex are entirely in the 5.8S rRNA sequence, and ES4 includes nucleotides of both 5.8S and 28S rRNA. The mRNA matches of these LSU rRNA segments were not evaluated in detail.

### Matching of rRNAs with antisense and sense tracts in mRNAs

Canonical and contiguous matches longer than 15 nucleotides are not found in CSL, ESS, and CSS, and are rare in ESL other than 7, 15, and 27 (see Table S5). The AU-rich matches of six nucleotides are not stable at physiological homeotherm temperatures (Kibbe, 2007; Mathews et al., 2007) and <6-nt matches of any nucleotide composition are quite unstable at 37°C. We therefore examined tracts of 7–15 nucleotides (nt) in ES and CS of rRNAs that match Watson-Crick antisense counterparts (the contiguous G:C and A:U matches, not including the G:U matches) of the same length in mRNA sectors (5′utr, cds and 3′utr), starting at position 1 in both sequences and shifting the match window by 1 nt until the remaining sequence length equaled (match length −1); e.g., the 801-nt ES7L was examined for 795 successive 7-nt matches. (Matches of 16–20 nucleotides were also scanned, and are enumerated in the Table S5.) The matching was done with Visual Basic macros in Microsoft Excel. Matching was also scanned for the sense counterparts, for a global comparison with the antisense matches (see Table 1). The sense matches may serve for competitive disbanding of mRNA folds and also of mRNA links to proteins.

**Table 1:** A summary of mRNA matching by expansion and core segments of 28S and 18S rRNAs.

**Group**	**Total nucleotides**	**Matches of 7-15 nucleotides**, × **10**^**−6**^				**Regression slopes**		
		**All sectors**	**5′utr**	**Cds**	**3′utr**	**5′ utr**	**Cds**	**3′utr**
**ESL** [15]	2,387	19.312	4.591	10.054	4.667	−0.3402	−0.4087	−0.4586
Matches per 100 nt		8,090	1,923	4,212	1,955	[0.0328]	[0.0341]	[0.0277]
**ESS** [16]	2,648	14.733	1.405	7.415	5.913	−0.5551	−0.5851	−0.5936
*as % ESL*		*76.3*	*30.6*	*73.8*	*127*	[0.0116]	[0.0031]	[0.0052]
Matches per 100 nt		5,564	531	2,800	2,233			
*as % of ESL*		*68.8*	27.6*	*66.5*	*114^&^*			
**CSL** [11]	521	2.783	0.3523	1.3713	1.059	−0.5204	−0.5631	−0.6069
*as % ESL*		*14.4*	*7.67*	*13.6*	*22.7*	[0.0023]	[0.0044]	[0.0096]
Matches per 100 nt		5,341	676	2,632	2,033			
*as % of ESL*		*66.0*	35.2*	62.5*	*104*			
**CSS** [12]	1,343	6.942	0.6758	3.508	2.758	−0.5365	−0.5748	−0.5569
*as % ESL*		*35.9*	14.7*	*34.9*	*59.1*	[0.01]	[0.0115]	[0.0235]
Matches per 100 nt		5,169	503	2,612	2,054			
*as % of ESL*		*63.9*	26.2*	62.0*	*105*			

*Numbers of segments are shown in brackets after the segment labels. For other details see the Methods section. Matches per 100 nt lower than those in ESL in Wilcoxon rank sum test at p < 0.01 are labeled with asterisks, and the one higher than ESL by an ampersand. Maximal fold difference from ESL match count is always highest with ESS (13.0, 7.33, and 4.41-fold for 5′utr, cds and 3′utr, respectively) and lowest with CSL (3.27, 1.36, and 0.789-fold for 5′utr, cds and 3′utr, respectively). For all three sectors, the ESL regression slope ± double SD does not overlap with slope of any other segment group, i.e., all ESL regressions are significantly less steep than those of other segment groups. Double standard deviations of the regression slopes are shown in brackets below the slope values. Some data rows or columns are rendered in italics to improve the readability*.

To obtain an insight as to the overall selectivity in the canonical matching of human mRNAs by the expansion segments of human 28S and 18S rRNAs, the successive 7-nucleotide tracts (starting, as above, at the 5′-terminus) of scrambled sequences of the expansion segments were matched with the three sectors of human mRNAs. This used 10 successive shuffles of each of the native ESL and ESS sequences generated by the Visual Basic function *scramble* (available from https://chandoo.org and also listed in the Table S6).

### The RNA secondary structure

Predictions of oligoribonucleotide and polyribonucleotide secondary structures were obtained with *RNAstructure* program (Mathews et al., 2007) and with *RNAfold* program (Gruber et al., 2008). These programs were also used for modeling the free energies of secondary structure formation/disbanding.

### RNA-protein binding

Parameters of nucleobase binding potential of protein amino acids listed in Table 5 of Jones et al. (2001) were used to evaluate protein-binding potential of RNA segments. The results generally corresponded with those of bindN+ program (Wang et al., 2010).

### Statistical testing

Non-parametric Wilcoxon signed rank (paired) test was used for characterization of paired data, and Wilcoxon/Mann–Whitney rank-sum test was applied to non-paired data. The Monte Carlo *t*-tests were also done in parallel to nonparametric tests. Differences with *p* < 0.01 were taken as significant. Linear regressions were characterized in Microsoft Excel 2010, using both built-in functions and Visual Basic macros.

## Results

### The expansion segments of 28S rRNA match mRNAs much more than those of 18S rRNA or the core rRNA parts

Examination of antisense matches to mRNA sectors in rRNA expansion or core segments was done for contiguous tracts of 7–15 nucleotides. This is similar to matching of microRNAs with mRNAs using “seed” segments, as performed in various programs (Wong and Wang, 2015; Rennie et al., 2016). However, we did not limit counting to any single tract within sequences of rRNA segments (see section Methods). Matches longer than 20 nt are extremely rare in the ES of human 28S and 18S rRNAs. Matches of 16–20 nucleotides are fairly frequent in ESL7, 15, and 27, at about 15,000 finds (see Table S5). These finds largely represent overlapping matches to 5′utr and cds in a limited number of mRNAs, and could reflect a specialization in ESL interaction with mRNAs, which we are studying currently. The 16–19 nt matches are very rare in other ESL (58 finds) or in any CSL, ESS, and CSS (with totals of 70, 20, and 54 finds, respectively; see Table S5); no matches longer than 19 nt were encountered in these rRNA segments.

**Figure 1:**
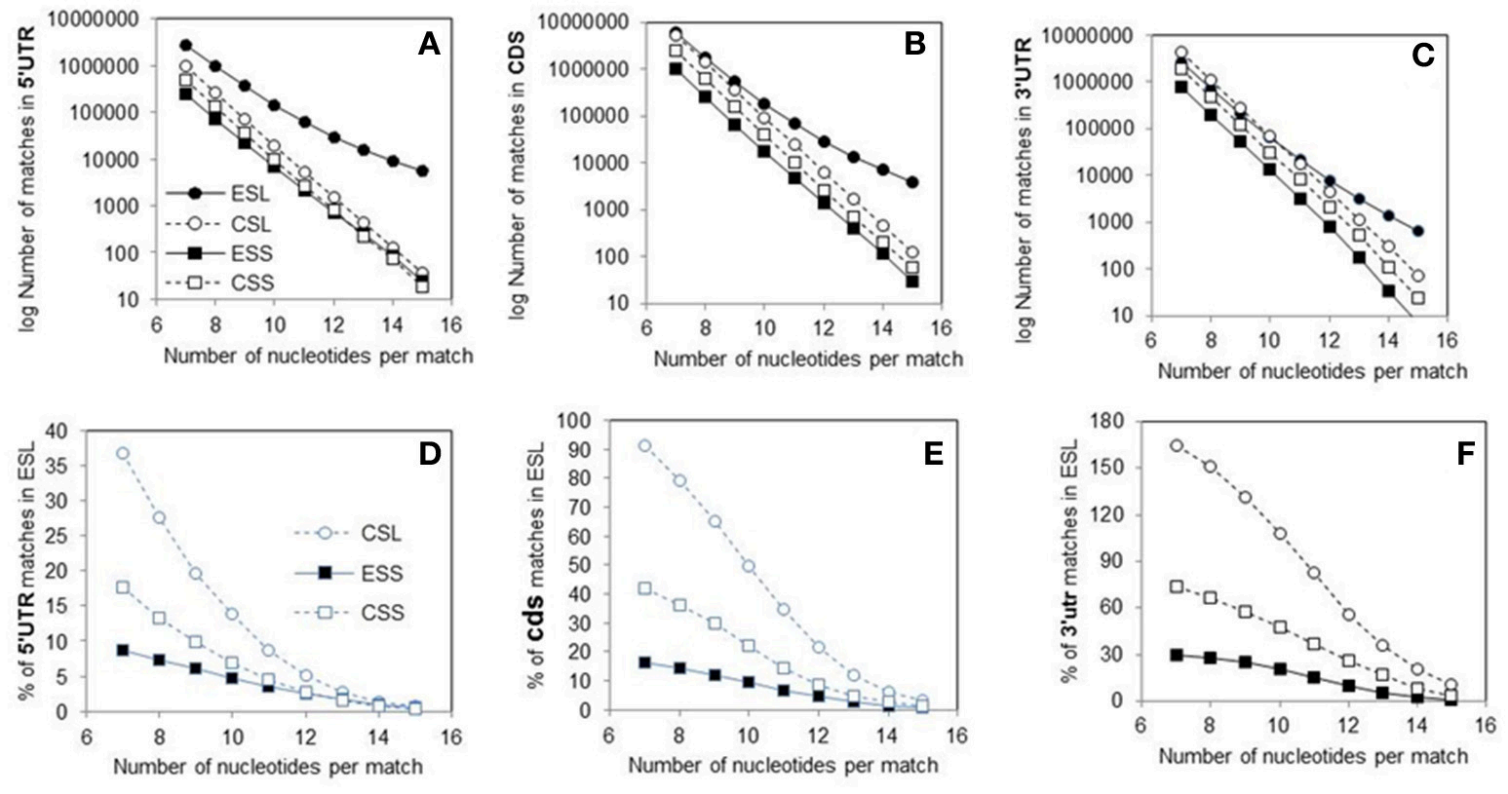
Counts of canonical contiguous 7–15 nucleotide matches of human rRNA segments with human mRNA sectors. **(A)** Matches in 5′utr. **(B)** Matches in cds. **(C)** Matches in 3′utr. Percentages of the numbers of matches relative to ESL are shown in graph **(D)** for 5′utr, in graph **(E)** for cds, and in graph **(F)** for 3′utr. In Wilcoxon signed-rank (paired) test at *p* < 0.01 the numbers of matches in ESL were higher than those in ESS for all three sectors, and also higher for 5′utr and cds. The percentage of matches relative to ESL tested as lower in 5′utr for all other rRNA segments, and in cds and 3′utr for ESS. The percentage of CSL matches relative to ESL in 3′utr however tested as higher. Slopes of the linear regressions on number of nucleotides in segment vs. log_10_ of match count for 5′utr, cds and 3′utr were: in ESL, −0.3402, −0.4087, and −0.4586; in CSL, −0.5551, −0.5851, and −0.5936; in ESS, −0.5204, −0.5631, and −0.6069; in CSS, −0.5356, −0.5748, and −0.6669. In linear regressions the ESL slopes were much lower than in other segment groups, with no overlaps at double *SD* (see Table 1). The *r*^2^-values were above 0.99 in all regressions.

The linear regressions (on number of nucleotides in segment vs. log_10_ of match count) of the 7–15 match counts for rRNAs and mRNAs are highly significant for all ES (*r*^2^ above 0.99). As evident from non-overlapping slope values and variances (see the caption of Figure 1 and Table 1), ESL with all mRNA sectors have highly significantly lowest rates of decrease in numbers of the matches with increase in size of the matching tracts.

The pooled matches to ESL in all sectors outnumber those in CSL by 23%, ESS by 75%, and CSS by 64% (Table 1). The number of matches is paramount in soliciting contact with long polynucleotide partners, and the large differences in this regard between ESL and other segments are strongly supported by paired Wilcoxon tests. (Interestingly, many mRNAs have multiple repeats of matches especially with ESL, in some cases with more than 20 repeats for the same 7-nt ESL tract.) The scores in other segment groups, while in most cases quite below ESL, differ sharply across mRNA sectors, in 5′utr being consistently much below ESL (69–92%), in cds strongly below ESL (26–84%), and in 3′utr either above ESL (by 27% in ESS), or below ESL (by 67% in ESS and 41% in CSS) (Table 1). The match frequency (or density) per 100 nt compared to ESL is very significantly lower for other 5′utr (65–74%) and quite lower for other cds (33–38%), but higher by 4–27% in 3′utr (and significantly for CSL; Table 1).

The 5′utr matches in the ES of 28S rRNA greatly outnumber those in other rRNA segments (Figure 1A and Table 1). This preponderance is already very large at the match length of 7 nt, and is increasing by up to three orders of magnitude in the length range of 10–15 nt (Figure 1A). The number of 5′utr matches per added nucleotide decreases by a factor of 1.6–2.2 at any ESL match length, a rate which is much less than for other groups. The decrease is in 2.7- to 3.7-fold range with all other groups (Figure 1A), with similarly uniform rates.

The cds matches (Figure 1B and Table 1) in ESL also outnumber those in other rRNA segment groups for all match lengths, but magnitude of the difference is less than for 5′utr (Figure 1E and Table 1). Above the length of 9 the ESL matches to cds outnumber those in ESS by more than one order of magnitude, and that also applies in comparison with CSS above 12 nt.

The 3′utr matches are somewhat above ESL for ESS at lengths of 7–9 nt, and then decrease below ESL uniformly in CSL (Figure 1C and Table 1). The CSS matches to this sector are distinctly less numerous compared to CSL, and the numbers of ESS matches are much below those in any other group (Figure 1C).

Matches in 5′utr and cds are below ESL for all other rRNA segments (Figures 1D,E). The difference is largest with ESS and increases uniformly with segment length.

The cores of 28S rRNA and all segments of 18S rRNA also have much lower numbers of long canonical mRNA matches than the ESL. Matches in 5′utr and cds are similar in numbers for ESS (Figures 1A,B). Large parts of the expansion segments that locate to ribosome surface apparently have no stable protein complement (Chandramouli et al., 2008; Ben-Shem et al., 2011) and could have a considerable potential for interaction with outside partners. This could apply especially to the ESL.

5.8S ribosomal RNA is highly folded and extensively associated with 28S rRNA by hydrogen bonding (see e.g., the supplement of Chandramouli et al., 2008). The potential for mRNA interaction in the unfolded sequence of this RNA is uniformly below 50% of the ESL potential (data not shown). Much of the folded 5.8S rRNA sequence as bound to the 28S rRNA molecule could be involved in operation of the ribosomal P site (Yin et al., 2003).

**Figure 2:**
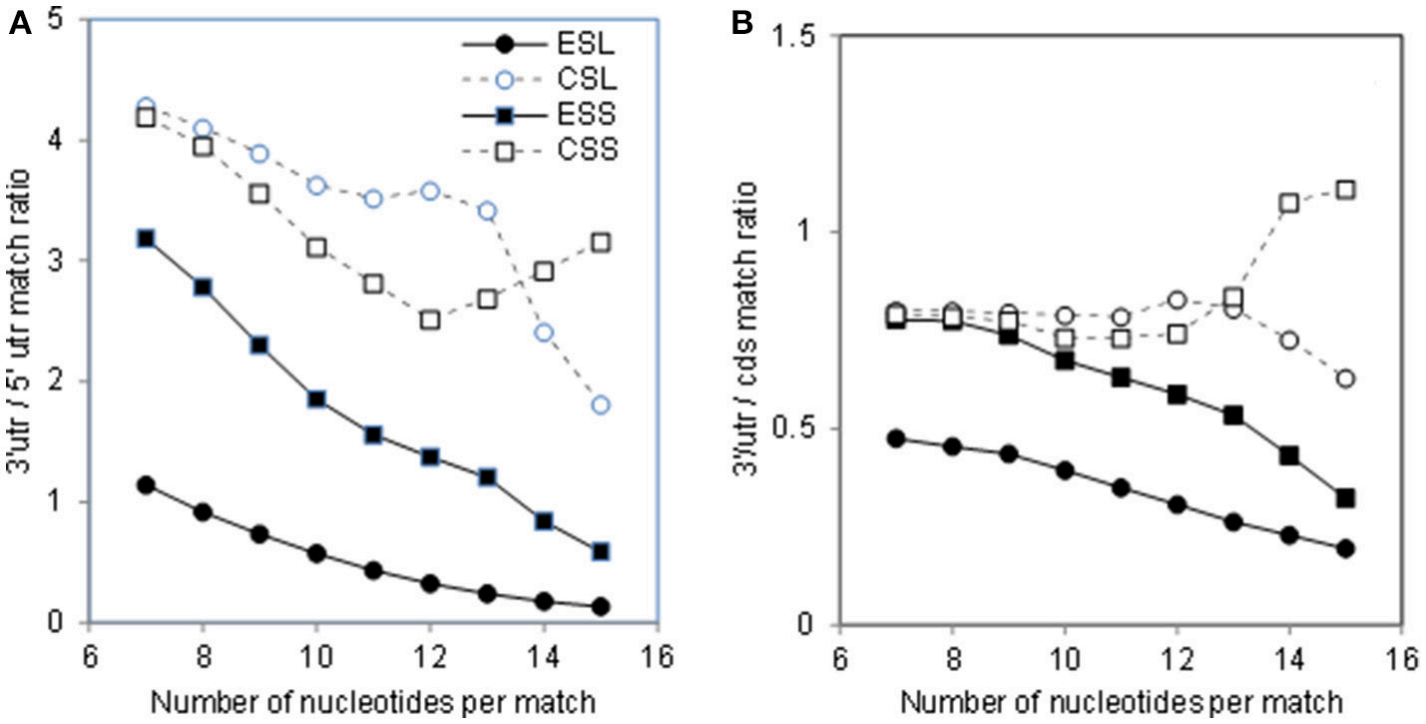
Ratios of rRNA matches in 3′utr to other mRNA sectors. **(A)** 3′utr/5′utr match ratios. **(B)** 3′utr/cds match ratios. In Wilcoxon paired test at *p* < 0.01 the 3′utr/5′ utr ratios were lower for ESL relative to all other rRNA segments. The 3′utr/cds ratios for ESS were lower than those for CSS and CSL. Both 3′utr/5′utr and 3′utr/cds CSL ratios were lower than those for the corresponding CSS segments. All differences with ESL were significant at *p* < 0.01 in Monte Carlo *t*-tests.

Abundance of the mRNA sector matches among rRNA segments could be compared via ratios of counts across match lengths (Figure 2). As seen in Figure 2A, the count ratio of 3′utr to 5′utr for all rRNA groups decreases almost linearly with match length up to 12 nt. The ESL 3′utr/5′utr ratios are above unity only for match length of 7, and decrease below 0.5 already at 11 nt, indicating a very large excess of 5′utr over 3′utr matches in long ESL matches (Figure 2A). The ratios for ESL7, ESL15, and ESL27 share the pattern of other ESL (data not shown). The ESS and CSS ratios are about 4 at 7 nt, and do not fall below 2 (Figure 2A). In ESL, 5′utr have more matches than 3′utr at 8–15 nt and much higher match density at any length.

The 3′utr/cds ratios (Figure 2B) for ESL and ESS are in the range of 0.5–0.8 for lengths of 7–10 nt, and then decrease slowly. The CSL and CSS 3′utr/cds match ratios remain in the range of 0.7–1 over the entire examined range (Figure 2B). Similar or larger mRNA interaction potential in ES cds compared to the respective 3′utr (Figure 2B) would support the use of ES in retrieval of mRNAs, e.g., by competitive displacement of mRNP proteins.

To get an insight about selectivity of the matching by rRNA expansion segments, we compared the numbers of mRNAs matched by the successive 7-nt tracts of the native ES with those in 10 successive randomly mixed sequences (see section Methods). For the ESL, the average difference was 0.72% (with 6.4% coefficient of variation), and for the ESS this difference was 3.6% (at 6.8% variation). This forecasted a low impact of random sequence permutation upon matching with short rRNA tracts. A detailed examination of this subject is outside of the scope of this work; however, as considered in the Discussion, this is expectable, and similar predictions are obtained with microRNAs.

### The density of mRNA matches is higher in ESL compared to other rRNA segments

**Figure 3:**
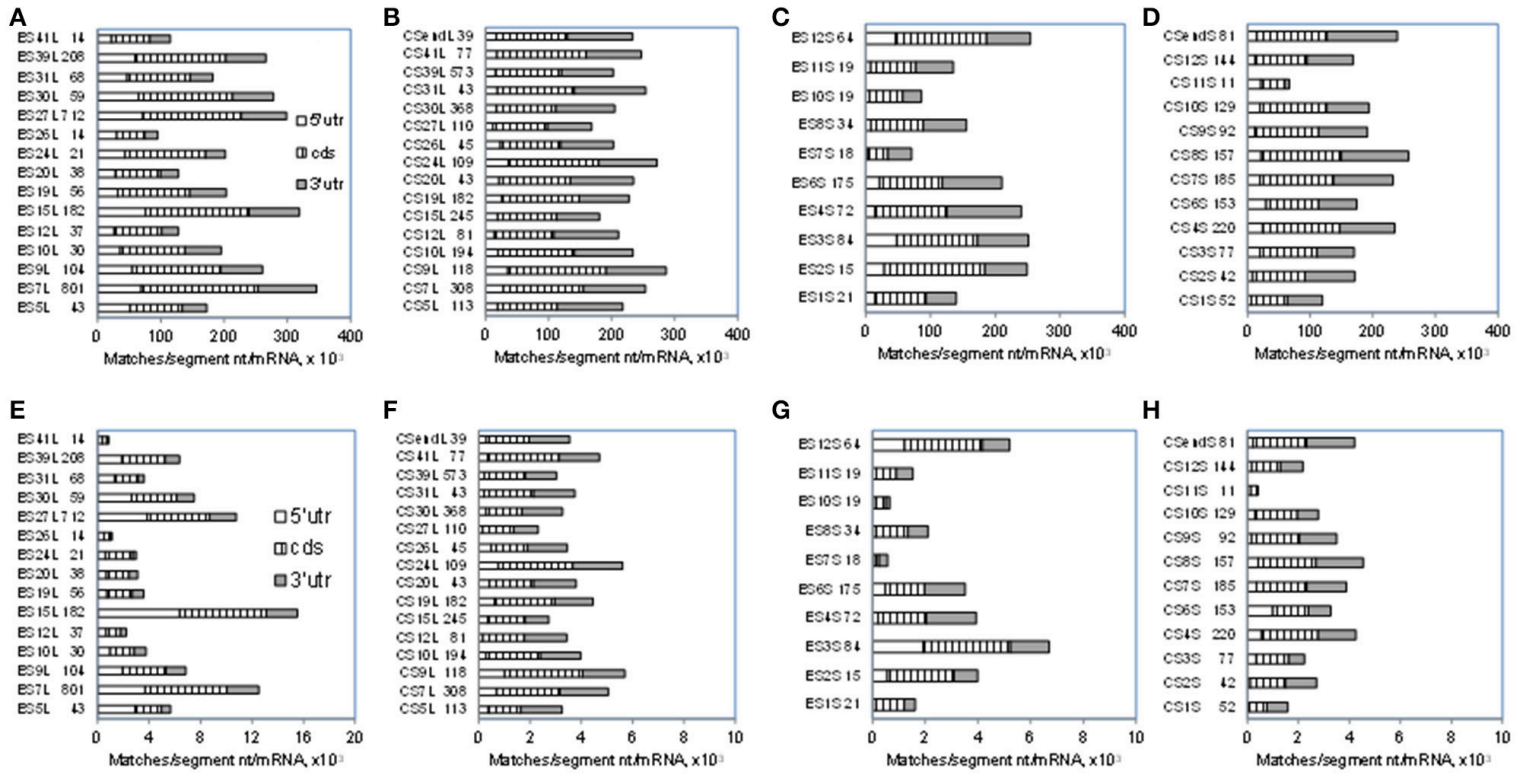
Densities of 7- and 10-nucleotide matches to human mRNA sectors in segments of human 28S and 18S rRNAs. Total numbers of matches in segments are expressed per number of nucleotides in the segment and per 1,000 mRNAs (see section Methods). Upper row, 7-nt matches: **(A)** ESL, **(B)** CSL, **(C)** ESS, **(D)** CSS. Lower row, 10-nucleotide matches: **(E)** ESL, **(F)** CSL, **(G)** ESS, **(H)** CSS. Numbers of nucleotides in the segments are listed next to the segment labels. Average numbers of nucleotides in the examined mRNA sectors are listed in Methods. For 5′utr, the ESL densities were at least two-fold larger than in other segment groups and also significantly higher in Wilcoxon rank sum test (WRST) against all other groups for either the 7- or the 10-nt match length. For cds segments there were no significant density differences in WRST. For 3′utr the ESL densities were for both match sizes lower than in CSL, with significant difference in WRST. The above density differences showed closely similar confidence levels in Monte Carlo *t*-tests.

As seen in Figure 1 and Table 1, numbers of mRNA matches in ESL are much above those in other rRNA segments. Somewhat similar profiles are found for match densities per segment nucleotide as expressed (for numerical convenience) per 1,000 mRNAs (Figure 3). Among 7-nt tracts (Figures 3A–D) ESL7, ESL15, and ESL27 have 300 or more matches per segment nucleotide in 1,000 mRNAs, and this is mostly due to cds and 5′utr contributions. (It should be noted that all rRNA segments of more than 50 nt have matches of 7 nt in >95% mRNAs; see Table S3) Densities of 7-nt matches are quite uniform in the core 28S rRNA segments, CSL (Figure 3B). With the 18S ES 7-nt matches (Figure 3C), short ESS2, ESS3, and ESS4 have higher densities than the long ESS6, and most ESS have densities above 200. The core 18S 7-nt segments generally have densities of 7-nt matches similar to ESS (Figure 3D).

With 10-nt matches (Figures 3E–H), the densities in large ESL7, ESL15, and ESL27 are very high compared to other rRNA segments (Figure 3E); across rRNA segments, only these ESL have more than eight 10-nt matches per segment nucleotide in 1,000 mRNAs, and ESL15 has about 15. Among LSU core segments, CSL24 has highest densities of both 7-nt (Figure 3B) and 10-nt (Figure 3F) matches. Interestingly, the large ESL39 has a quite low 10-nt match density (Figure 3E) and the largest ES of 18S rRNA, ESL6, has that density below several other ESS (Figure 3G). Among 10-nt matches for core 18S rRNA segments (Figure 3H) CSS1 and CSS11 have the least, and CSS4, CSS8, and CSSend the highest densities. The highest densities for both match lengths are found for large ESL 7, 15, and 27 (Figures 3A,E).

Matching with mRNAs was also examined for 5.8S rRNA. This RNA is intricately associated with 28S rRNA and thus may not significantly interact with mRNAs. The mRNA match density for free unfolded 5.8S RNA is only 49% of that for ESL7, and about equal to average of the 28S core segments (data not shown).

### The GC content of mRNA matches is much higher in ESL than in other rRNA segments

The ESL show at least 21 percentiles of GC above other rRNA segments (which are quite close in that content, averaging 56–58% GC) (Table 2). As seen in Table 2, the GC content of mRNA matches to ESL segments is also very much higher than in other rRNA segments. This is found for all mRNA sectors, and the difference is largest with 3′utr; this is seen for both 7- and 10-nt matches, and the latter show uniformly larger differences across rRNA segments and mRNA sectors. ESL matches also have higher GC content than the full ESL sequences. The largest GC content in matches is for all groups found with 5′utr, and the lowest with 3′utr matches. With full sequences of matched mRNA sectors the difference is much smaller but still present. The GC content of 3′utr matches is very much above that of the respective full sector sequences, indicating a strong selection of GC-rich elements across relatively low-GC 3′utr sequences. The ESL matches in 5′utr have >90%, in cds >84%, and in 3′utr ~80% GC. The difference in GC content of matches between 5′utr and other sectors was highly significant in nonparametric as well as the Monte Carlo testing for all lengths between 7 and 15 nt, while this content did not differ significantly among CSL, ESS, and CSS tracts. The mean ESL GC content was very significantly higher than those of other groups, while the CSL, ESS, and CSS mean GC contents did not differ significantly.

**Table 2:** GC content of 7-nucleotide and 10-nucleotide mRNA matches of rRNA segments.

**Group**		**Antisense matches**			Sector sequences matched*		
	**Pooled segment GC%**	**5′utr GC%**	**cds GC%**	**3′utr GC%**	5′utr GC%*	cds GC%*	3′utr GC%*
**7-nt matches**							
ESL	79.15	90.98	84.12	79.86	69.31	58.65	49.87
CSL	56.45	69.9	59.01	49.29	63.75	53.8	43.83
ESS	58.28	78.77	61.95	44.16	66.23	54.16	43.19
CSS	56.62	72.35	60.40	49.78	64.21	54.02	43.84
**10-nt matches**							
ESL	79.15	93.17	87.17	81.33	71.47	60.6	51.61
CSL	56.45	72.45	59.64	49.77	65.06	54.3	44.34
ESS	58.28	86.26	68.78	44.66	69.46	55.87	43.66
CSS	56.62	75.97	62.15	52.12	66.07	54.92	44.69

*Items in columns with asterisks are the averages of GC content of mRNA sectors that have rRNA matches. Most of the involved sectors have multiple rRNA matches. Average GC% in sectors of human mRNAs tested (18,810): 5′utr, 63.25; cds, 53.20; 3′utr, 44.26. For both the matches of 7 and 10 nt, all ESL means are significantly above the respective means of other sectors at p < 0.001 in Wilcoxon rank sum tests; none of the means of CSL, ESS, and CSS test significantly against each other. The same outcomes were obtained in Monte Carlo t-tests*.

### ES7, ES15, and ES27 of human 28S rRNA are much longer than in non-hominid eukaryotes

Size and GC content of five largest ESL across eukaryotic 25-28S LSU RNAs are presented in Table 3. (The list of rRNA sequence addresses is available in Table S1.) Pooled nucleotides of these segments amount to almost 40% of the entire sequence of human 28S rRNA (Table 3). This fraction descends steeply down the phylogenetic ladder (Table 3), with the fish fraction being 0.55, the nematode 0.46, and the alveolate only 0.38 of the human. In many cases a similar definition of the segments is achieved using *Saccharomyces cerevisiae* 25S rRNA as the template (Parker et al., 2015).

**Table 3:** Size and GC content in five large expansion segments of eukaryotic 25–28S rRNAs.

**Group and number of species**		**ES7**	**ES9**	**ES15**	**ES27**	**ES39**	***GC% for pool of large ES***	***Core GC%***	**Large ES pool as % sequence**
Human [1]	#nt	801	104	182	712	208			39.9
	*GC%*	*83.8*	*79.8*	*84.1*	*86.5*	*82.7*	*84.5*	*56.2*	
Hominid ape [3]	#nt	779 ± 28.0	105 ± 0.58	159 ± 32.1					
	*GC%*	*83.5 ± 0.40*	*80.3 ± 0.61*	*81.1 ± 2.94*					
Rodent [2]	#nt	662 ± 60.8	105 ± 2.12	131 ± 7.07	615 ± 12.0	213 ± 5.66			36.3
	*GC%*	*81.8 ± 1.24*	*79.9 ± 0.95*	*84.4 ± 2.94*	*82.6 ± 1.04*	*77.71 ± 1.07*	*81.7 ± 0.29*	*55.9 ± 0.14*	
Bovine [2]	#nt	600 ± 65	105 ± 0.51	87 ± 10	592 ± 73	181 ± 0.50			34.4
	*GC%*	*70.6 ± 0.35*	*67.9 ± 4.5*	*65.1 ± 1.2*	*73.2 ± 1.9*	*65.4 ± 1.3*	*70.6 ± 0.65*		
Avian [1]	#nt	729	104	22	448	140			32.5
	*GC%*	*86*	*82.7*	*81.8*	*87.5*	*80.7*	*85.7*	*57*	
Amphibian [1]	#nt	442	124	24	324	133			25.7
	*GC%*	*85.7*	*88.7*	*75*	*84.3*	*81.2*	*84.8*	*55.8*	
Fish [6]	#nt	404 ± 19.6	98.4 ± 1.08	24.8 ± 1.88	230 ± 12.9	85.3 ± 42.2			22.3
	*GC%*	*70.9 ± 3.4*	*78.3 ± 1.75*	*73.0 ± 2.0*	*72.6 ± 2.51*	*61.1 ± 2.7*	*73.2 ± 1.48*	*54.9 ± 0.78*	
Chordate [2]	#nt	325 ± 9	96 ± 2	33.5 ± 3.5	142 ± 32	121.5 ± 8.5			20.1
	*GC%*	*72.8 ± 0.29*	*74.0 ± 0.54*	*67.1 ± 0.45*	*69.8 ± 0.23*	*62.2 ± 0.65*	*70.3 ± 0.66*	*52.8 ± 0.1*	
Mollusk [1]	#nt	310	95	24	175	142			20.4
	*GC%*	*62.9*	*67.4*	*66.7*	*65.7*	*54.9*	*62.7*	*53.7*	
Insect-1 [5]	#nt	404 ± 19.6	98.4 ± 1.08	24.8 ± 1.88	230 ± 12.9	85.3 ± 42.2			21.2
	*GC%*	*70.9 ± 3.38*	*78.4 ± 1.75*	*73.0 ± 2.01*	*72.6 ± 2.51*	*61.1 ± 2.7*	*62.3 ± 1.48*	*54.0 ± 0.82*	
Insect-2 [3]	#nt	300 ± 14.2	109 ± 5.81	48.7 ± 0.88	169 ± 30.9	126 ± 52			19.3
	*GC%*	*31.2 ± 4.3*	*33.8 ± 2.04*	*18.5 ± 0.34*	*34.1 ± 1.61*	*37.8 ± 6.09*	*31.5 ± 1.51*	*40.7 ± 0.28*	
Nematode [1]	#nt	213	101	22	177	133			18.5
	*GC%*	*55.4*	*57.4*	*59.1*	*52.5*	*53.4*	*54.6*	*47.7*	
Fungal [7]	#nt	194 ± 4.0	67.1 ± 1.86	28.3 ± 4.6	161 ± 6.9	119 ± 3.7			16.9
	*GC%*	*57.9 ± 2.6*	*54.2 ± 1.3*	*53.1 ± 4.2*	*61.7 ± 2.4*	*51.2 ± 2.7*	*56.8 ± 2.05*	*49.8 ± 1.01*	
Alveolate [2]	#nt	204 ± 1	69.5 ± 0.5	17 ± 3	137.5 ± 0.5	72.5 ± 21.5			15.0
	*GC%*	*48.3 ± 0.97*	*56.8 ± 1.13*	*50*	*42.9 ± 0.57*	*29.8 ± 4.28*	*43.6 ± 0.17*	*45.1 ± 0.1*	
Angiosperm [4]	#nt	179 ± 3.35	65.3 ± 1.18	14	163 ± 0.58	127 ± 2.21			16.2
	*GC%*	*75.9 ± 3.06*	*65.1 ± 1.30*	*66.1 ± 1.79*	*75.6 ± 1.95*	*73.9 ± 4.73*	*73.5 ± 2.68*	*53.9 ± 0.29*	

*Data for numbers of nucleotides (# nt) and for GC% are means with standard errors. The number of species analyzed is shown in brackets after the group labels. Note that the estimates of pooled GC% and % sequence for the large ES refer to combined nucleotides of the five segments. Core GC% refers to pooled core segments of the entire sequences. The segment boundaries were defined from alignment to those of human 28S rRNA (see section Methods). The size and GC% of 25-28S rRNAs are shown in Table S1. The reported sequences of bovidal 28S rRNAs are 8–10% shorter than in other mammals (see Table S1), and this also applies to ESL7, while the size of bovidal ESL15 is 66% of the rodent, and only 48% of the human (This table). The GC contents of whole sequences and of large ESL are, respectively, about 10 and 14 percentage units below averages of other mammals. The GC differences point to a lower-GC profile, somewhat similar to that in Insect-2 group. Some data rows or columns are rendered in italics to improve the readability*.

As seen in Table 3, ESL15 and ESL27 are quite longer (at a very similar high GC content) in human 28S rRNA compared to other mammals, and much longer than in other vertebrates. The ESL7 is very long in all homeotherm vertebrates, but appears to be distinctly the largest in human 28S rRNA. This segment is much shorter in poikilotherm vertebrates, plants, and lower eukarya (Table 3). The ESL39 is also rather longer in homeotherm vertebrates and has a much smaller (and similar) size across other metazoans. The ESL9 is of similar size across metazoans and quite short in lower eukarya and in plants. Most of the difference in size is seen for ESL15, which appears to have expanded radically in the mammal, and even further in the hominid. This segment is comparatively very short in plants, which also applies to ESL7 and ESL27. The next section presents a more detailed characterization of ESL15 across eukaryotic species.

The overall GC content of the ES segments is uniformly above 80% in tetrapod vertebrates, and in the range of 60–70% in poikilotherm metazoans including the fish. However, in some insects there is an inversion of the ESL nucleotide composition bias in favor of AU (Table 3). Most metazoan core segments have much lower GC content than the human, by 20–25 GC percentiles; the insect rRNAs with AU bias in the ESL have distinctly lower core AU percentile. The 25S rRNAs of lower eukarya have quite low GC content in the ESL, with little or no distinction from the core segments. Interestingly, plant 25S rRNAs with short ESL show a high GC bias in these segments. Overall, high nucleotide bias in the ESL is found in taxonomic units that have multiple cell types and large organismic complexity.

### ESl15 is by far the most expanded in mammals, with the largest increase in hominids

**Figure 4:**
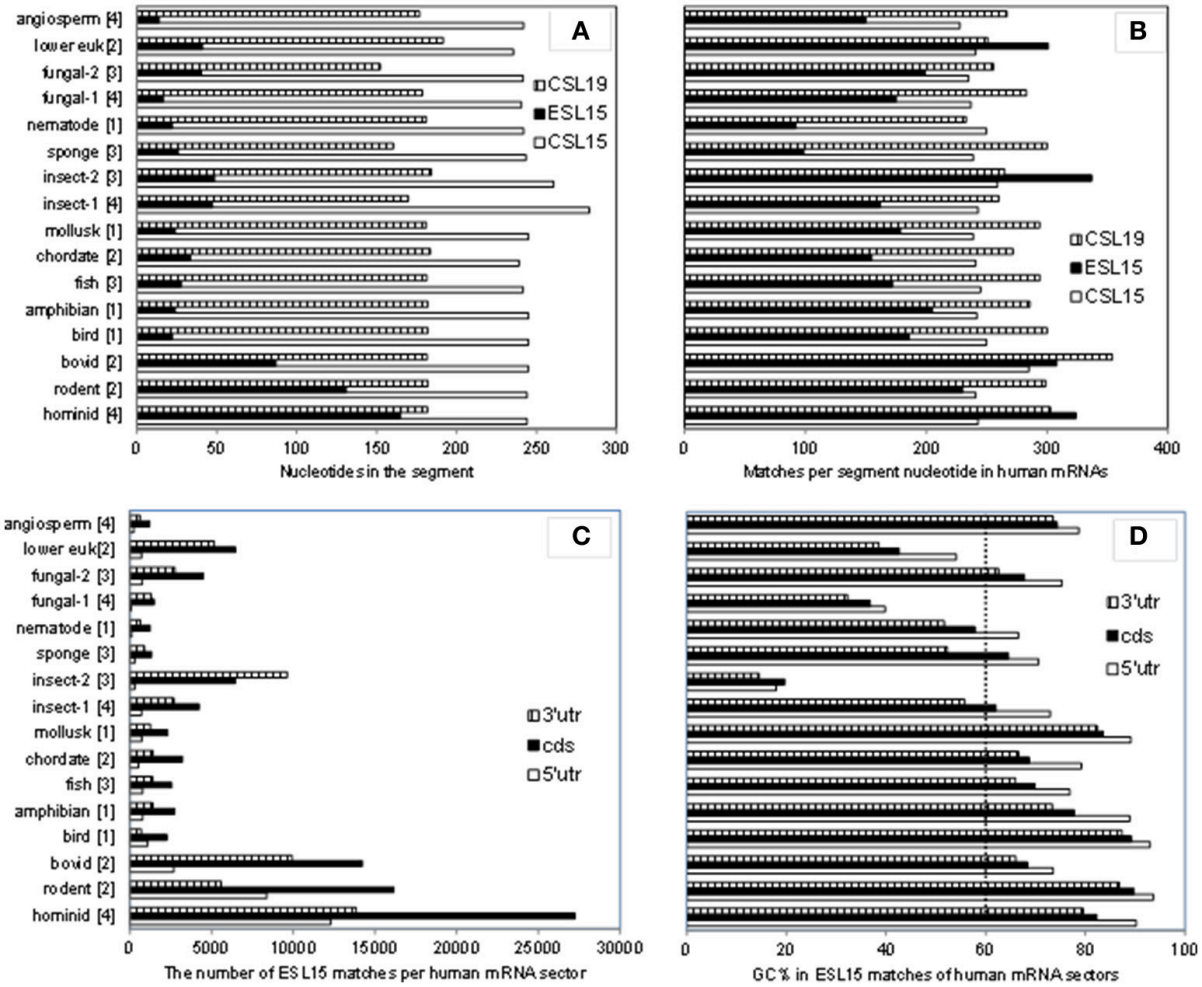
A comparison of size and GC content in ESL15 and the neighboring core segments of 25-28S rRNAs across the phylogenetic ladder. **(A)** Average number of nucleotides per CSL15, ESL15, and CSL19 segment. **(B)** Average number of 7-nt human mRNA matches per CSL15, ESL15, and CSL19 segment. **(C)** Overall 7-nt human mRNA matches by ESL15 segments. **(D)** Average GC content of 7-nt ES15L antisense matches in human mRNAs. Average GC contents (±*SD*) for entire segment sequences in all groups are: 54.9 ± 4.2 (46.6–60.4) for CSL15, 62.7 ± 16.6 (18.5–84.4) for ESL15, and 55.6 ± 5.1 (45–62.6) for CSL19. For details about the examined rRNAs see the Table S1.

As shown in Table 3, ESL15 is short in all non-mammals, shows a large size increase over non-mammals even in its shortest (bovid) mammalian sequences, and is very much expanded in hominids. The definition of this segment via alignment with human 28S rRNA appears to be adequate in view of the fact that the flanking large core segments in all eukaryote 25–28S rRNAs have quite similar length (Figure 4A and Table S4) and GC content (legend of Figure 4 and Table S4). ESL15 is at least six-fold larger in the hominid compared to the non-mammalian average (Figure 4A), with an increase in GC content of at least 40% over invertebrate metazoans (Table 3). The density of ESL15, CSL15, and CSL19 matches in human mRNAs is not much different between vertebrates or invertebrates (Figure 4B). However, the number of matches per human mRNA sector (Figure 4C) is much higher for hominid ESL15 vs. any other ESL15 (and also significantly higher in Wilcoxon rank-sum testing, in as much that testing is meaningful in this case). The 3′utr matches of ESL15 in several invertebrates and in lower eukarya have below 60% GC (Figure 4D). Irrespectively of ESL15 size, the density of human mRNA matches is similar for all available land vertebrates (including a poikilotherm species) and decreases in aquatic poikilotherm vertebrates (Figure 4B). This density is also much lower in the short ESL15 of invertebrates and plants. On the other hand, the density of human mRNA matches is across species quite similar for the large core segments preceding and following ESL15 (Figure 4B). These segments as expected are highly similar in size, with <6% variation across the examined eukaryote 25-28S rRNAs, as opposed to 90% for ESL15 (Table S4), and also do not differ much in GC content across eukarya (Table S4).

### Among rRNA segments, ESLs could also have the largest potential for interaction with proteins

Due to the much higher GC and G content than other groups of rRNA segments (see Table 3), ESLs could be expected to have higher potency for interaction with proteins (see Jones et al., 2001; Biot et al., 2004; Ellis et al., 2007 for the affinities of protein amino acids for nucleobases and the backbone ribose and phosphate). For mRNPs associated with the ER (Cui and Palazzo, 2014; Reid and Nicchitta, 2015) or with other subcellular networks (Jansen, 1999) this may aid a competitive detachment of the mRNP protein component prior to mRNA entrance in ribosome′s translation tunnel (Zimmermann et al., 2016). To obtain a rough estimate of the interactive potential with proteins, segments of 28S and 18S rRNAs were examined for frequencies of H bonding and of van der Waals interaction of the nucleobases with protein amino acids (calculated using parameters from Table 5 in Jones et al., 2001). The estimates are presented in Table 4.

**Table 4:** Predicted frequencies of hydrogen bonding and van der Waals interaction with protein amino acids for nucleobases in segments of human 28S and 18S ribosomal RNAs.

	**% Hydrogen-bonding frequency**						**% van der Waals frequency**					
**Group**	**# nt**	**% GC**	**G**	**C**	**A**	**U**	***G + C***	**G**	**C**	**A**	**U**	***G + C***
ESL	2,387	79.2^*^	52.5^*^	32.9^*^	5.53^x^	9.06^x^	*85.4*	51.5^*^	28.7^*^	9.13^x^	10.7^x^	*80.2*
CSL	2,648	56.5	42.6	22.8	20.7	13.8	*65.4*	37.0	17.7	30.8	14.5	*54.6*
ESS	527	58.3	38.9	28.7	12.1	20.2	*67.7*	35.6	23.6	18.7	22.1	*59.2*
CSS	1,343	56.6	44.6	22.9	13.4	19.2	*67.5*	40.4	18.6	20.4	20.6	*59.0*

The proposed frequencies of H bonding and van der Waals interaction with 20 protein amino acids for the nucleobases (Jones et al., 2001) were summed for individual nucleotides in segments of 28S rRNA (ESL and CSL) and 18S rRNA (ESS and CSS), and then tabulated as percentages of the respective sums. #nt, the number of nucleotides in all segments of a group.

* *Means above all other groups, ^x^means below all other groups at p < 0.01in Wilcoxon rank sum tests. Some data rows or columns are rendered in italics to improve the readability*.

Both the potential H-bonding and van der Waals protein-associating contributions of ESL guanine and cytosine are very significantly above those in other segment groups, while the ESL adenine and uracil contributions are much below other segments in overall averages and in Wilcoxon/Mann–Whitney rank sum testing (Table 4). This is reflected in GC contents of the respective segment groups, which for ESL segments are very significantly above other groups in both the actual means and in rank sum tests (Table 4). As seen in Table 4, ESLs would have by far the largest GC contributions to protein interactive potential among rRNA segments. Other segments in both rRNAs are not significantly different in frequency contributions, as the (G + C) frequency sums are quite close for H-bonding, and fairly close for van der Waals frequencies (Table 4). It should be noted that the capacity for either hydrogen-bonding or van der Waals interaction via the backbone ribose and phosphate, while somewhat larger than for the nucleobases, could be roughly similar for the four main RNA nucleotides (Jones et al., 2001; Ellis et al., 2007; Zirbel et al., 2009).

## Discussion

It should be reiterated that in the mature ribosome most of the core and much of the expansion sequence of eukaryotic rRNAs is not extensively available for interaction with extraneous RNAs. However, significant portions of the ES are not stably masked *in situ* (Chandramouli et al., 2008; Armache et al., 2010; Ben-Shem et al., 2010, 2011; Klinge et al., 2011) and could be contacted by extraneous RNAs and proteins. The ES thus present sequences that have significant outward accessibility and could experience canonical matching via loops, as well as the super-imposed Hoogsteen matching (Holland and Hoffman, 1996) and the triple-strand matching (Dinman et al., 2002) via stems. Canonical matches of up to 11 nucleotides, which predominate in single-stranded RNA folding, have low melting temperatures and should be structurally quite dynamic (see also Gupta et al., 2013). Also, acidic 60S proteins have helicase motifs, and initiation factors with very similar acidic motifs have helicase activity (Parsyan et al., 2011; Hull and Bevilacqua, 2016). These proteins could enhance the single-stranded availability of any encountered RNA regions.

Selectivity in matching of the rRNA expansion segments with mRNAs appears to be generally low, and is little changed by sequence scrambling. The principal difference between ESL and other rRNA segments is in the number of repeated matches per mRNA sequence. Selectivity of the canonical matching of human mRNAs by human microRNAs is known to be relatively low even if examined only for the “seed” segments (nucleotides 2–8) of microRNAs and for the 3′utr of mRNAs. The numbers of miR “seeds” matched by 3′utr typically are about 200 (as can be assessed in programs by Wong and Wang, 2015; Rennie et al., 2016), but could be as high as 1,700 per 3′utr, and average 15.7 miRs per 100 3′utr nucleotides. The matching of successive miR tracts shifted by a single nucleotide, as performed in Parker et al. (2016) and in this work with rRNA segments, addresses more mRNAs and especially augments numbers of repeated matches. An in-depth examination of selectivity (which is outside the scope of the present study) would of course require, beside the *in silico* work, examination of the binding of specific oligonucleotides and polynucleotides employing e.g., the techniques of nuclease digestion, gel chromatography and electrophoresis, ultracentrifugation, and immunoprecipitation.

It is important to note that guanine figures prominently in non-canonical base pairing and would support that type of pairing even as embedded in helices (Holland and Hoffman, 1996; Nagaswamy et al., 2000). This may apply to both canonical and non-canonical triple helices (Mizuta et al., 2005; Mathews and Case, 2006). The abundant G homoiterons of ESL (Parker et al., 2015) could also be significantly involved. Large hairpins can serve in triple helix RNA formations (Yu et al., 2011). The remarkable 16S rRNA triple helices involving multiple homoiterons (Nagaswamy et al., 2001) could lend support regarding the association between 28S rRNA ES stems or open tracts (especially those with long G or C homoiterons; Parker et al., 2015) and mRNAs in mRNPs. Viral triple-helical pseudoknots are important in control of viral RNA translation (Michiels et al., 2001); triple helices may form between mRNAs in mRNPs and large homoiteron- and GC string-rich ES of 28S rRNA (what, however, could be labor-intensive to study).

Both helical and single-stranded parts of ES could compete with mRNAs for protein components of mRNPs to facilitate their separation. Interaction of ES parts with mRNAs and/or mRNP proteins might help entrance of mRNA into the mRNA tunnel of the ribosome (Zimmermann et al., 2016). The extraction of mRNAs from mRNPs by rRNA ES does not have to discriminate between mRNA sectors; based on both the GC content and density of the matches, the release of 5′utr could occur preferentially. Assuming only 5% of ESL as dynamically available in single-stranded form on LSU surface facing mRNP complexes in the membrane, there could be more than 50 ESL matches per mRNA (see Table 1). Evolution of ESL size and GC content (Table 3) could be largely responsible for the GC enrichment seen in LSU rRNA phylogeny (Mallatt and Chittenden, 2014).

Results presented in Figure 4 and Table 3 indicate that ESL15 only became prominent in mammals, and may have expanded significantly even between rodents and hominids. The strong similarity of human mRNA match density across mammalian ESL15 would support a mass-action matching that depends on size and GC content of the segment more than on specific (and hardly on unique) motifs. Large similarity in the respective sizes of core segments CSL15 and CSL19 across eukarya (with quite similar GC contents; Figure 4) would also be in favor of a mammalian-specific evolution of ESL15.

Numerous studies indicate considerable, and even principal, cytoplasmic association of mRNAs with intracellular membranes, including both the ER (Lande et al., 1975) and the cytoskeletal system (Zambetti et al., 1990; Jansen, 1999; De Lucas et al., 2014). Messenger RNAs could be in mRNP granules, the processing bodies (“P-bodies”; Villacé et al., 2004; Brengues et al., 2005); mRNPs are also found in the ER of invertebrates (Wilhelm et al., 2005). ESL27 and ESL15, which are largely oriented toward the ER-facing backside of LSU (Chandramouli et al., 2008; Ben-Shem et al., 2011) may match mRNA nucleotides to facilitate removal of the mRNP protein and help transfer of mRNA to the mRNA tunnel.

Expansion segments of mammalian 28S rRNAs are largely located toward surface of the LSU, with considerable parts not stably associated with ribosomal proteins (Larsson and Nygård, 2001; Chandramouli et al., 2008). To a lesser extent this also could apply to 25S rRNAs of lower eukarya (Ben-Shem et al., 2011). Parts of the large LSU RNA that have dynamic contacts with partners could be large in the expansion segments of hominids, some of which are considerably larger than in other mammals or in a bird, and much larger than in poikilotherm vertebrates (Table 3). This could especially apply to ESL15 and ESL27 (which both are expanded considerably in hominids compared to rodents), and in particular to ESL15 (Table 3). The very large expansion of ESL15 in the mammal could indicate an important but unexplored gain in function. The abundant G and C homoiterons in the large ESL15 (Parker et al., 2015), a dynamic and solvent-exposed segment (Larsson and Nygård, 2001), might conceivably be involved in mobilization/extraction of mRNP-contained mRNAs that have homoiteronic repeats. This may involve both RNA-protein and RNA-RNA association.

The large expansion segments possess extensive areas not stably associated with proteins, which could be maintained by deformations induced by encounters with proteins that act as helicases. The cytoplasmic completion of the maturation of ribosomal subunits, with release of non-ribosomal proteins (Zemp and Kutay, 2007), could expose or create unstructured tracts. Initiation factors, which unravel mRNA stems (Marsden et al., 2006), may also act upon rRNA ES helices and stems.

Storage, mobilization and disposal of mRNAs currently are viewed mostly as confined to 18S rRNA, but from the present data obviously could admit roles for the very large (and largely not stably associated with ribosomal proteins, Chandramouli et al., 2008; Ben-Shem et al., 2011) ESL7, 15, and 27. The low numbers and much lower density of longer mRNA matches in ESS compared to ESL could point toward an expectable lack of substantial role for 18S rRNA in retrieval of mRNAs from mRNPs. These mRNPs should be mostly associated with intracellular matrices, and therefore could be much more accessible to ESL than to ESS. Due to high G content, the ESL should have strong preference over other rRNA segments for binding to ion-rich proteins (Shimoni, 1995 #1943), which would include the initiation/elongation factors. Within the mRNA translational curriculum, the ESL among rRNA segments appear to be the best equipped to chaperone the mobilization of mRNAs from mRNPs. However, the ESL matches, including the very numerous long matches, could also function in mRNA guiding and positioning, which deserves study. Additional *in silico* examination of mRNA/ES interaction should be helped by modeling, which is however not in the scope of the present description of the basal canonical interactivity of rRNA expansion segments with mRNAs.

## Author contributions

All authors performed literature searches and data derivation and calculation, and participated in interpretation and discussion of the results. SLP wrote the final manuscript.

### Conflict of interest statement

The authors declare that the research was conducted in the absence of any commercial or financial relationships that could be construed as a potential conflict of interest.

## Abbreviations

ES: rRNA expansion segment
ESL: expansion segment of 28S rRNA
CSL: core segment of 28S rRNA
ESS: expansion segment of 18S rRNA
CSS: core segment of 18S rRNA
LSU: large cytoplasmic ribosome subunit
SSU: smaller cytoplasmic ribosome subunit
mRNP: a complex of mRNA with protein(s)
nt: nucleotide
snoRNA: small nucleolar RNA.
