# Supplementary material for: The Expansion Segments of 28S Ribosomal RNA Extensively Match Human Messenger RNAs
Source: Front Genet. 2018 Mar 7;9:66. doi: 10.3389/fgene.2018.00066 (PMC5850279; doi:10.3389/fgene.2018.00066)
Supplement: Supplementary file 1 [file Table1.PDF]

Table S1 List of the examined ribosomal RNAs

GC% = sum of the percentages of G and C nucleotides in the sequence
#nt = the number of nucleotides in the sequence

Group	Entrez label	Organism	#nt	GC%
hominid-human	GI:555853	Homo sapiens	5035	69.18
hominid-ape	M30951.1	Gorilla gorilla gorilla	2467	70.17
hominid-ape	M30950.1	Pan troglodytes	2512	70.1
hominid-ape	M30952.1	Pongo pigmaeus	2487	69.76
rodent	GI:120444900	Mus musculus	4730	66.62
rodent	GI:374429576	Rattus norvegicus	4786	67.38
bovine	GI:83321215	Bos taurus	4542	58.59
bovine	JN412502.1	Bubalis bubalus	4552	58.17
avian	KT445934	Gallus gallus	4441	68.03
amphibian	GI:65056	Xenopus laevis	4082	65.39
fish	JN628435.1	Cyprinus carpio	4093	63.5
fish	U34336.1	Latimeria chalumnae	3410	61.03
fish	AF061798.1	Petromyzon marinus	4462	62.22
fish	AF061799.1	Hydrolagus colliei	3855	60.99
fish	U34337.1	Lepidosiren paradoxa	3471	57.74
fish	U34339.1	Protopterus aethiopicus	3355	57.17
chordate	X53538.1	Herdmania momus	3566	57.12
chordate	AF212177.1	Ciona intestinalis	3588	57.02
mollusk	AF342798.1	Placopecten magellanicus	3657	53.71
insect-1	L22060.1	Aedes albopictus	4102	51.76
insect-1	L78065.1	Anopheles albimanus	4022	54
insect-1	KC177663.1	Anopheles gambiae	3440	54.51
insect-1	AF463459.1	Samia ricini	4077	55.7
insect-1	AY210843.1	Tenebrio sp. JMM-2003	4457	56.32
insect-2	X99212	Chironomus tentans	3973	41.83
insect-2	M21017.1	Drosophila melanogaster	3900	39.28
insect-2	AF403819.1	Simulium sanctipauli	3734	40.44
nematode	X03680.1	Caenorhabditis elegans	3509	48.99
fungal	GI:671812	Candida albicans	3442	47.59
fungal	AF113137.1	Eremothecium gossypii	3390	48.02
fungal	AB026819.1	Magnaporthe grisea	3339	52.53
fungal	DQ888227.1	Saccharomyces cerevisiae	3396	47.94
fungal	Z19136.1	Schizosaccharomyces pombe	3497	47.04
fungal	AF510496.1	Talaromyces verruculosus	3285	52.91
fungal	AF510497.1	Trichoderma reesei	3271	52.4
alveolate	GI:10782	Tetrahymena pyriformis	3341	44.69
alveolate	GI:345541658	Tetrahymena thermophila	3354	44.75
angiosperm	GI:16131	Arabidopsis thaliana	3375	55.73
angiosperm	GI:169818	Oryza sativa	3377	59.43
angiosperm	AY049041.1	Triticum aestivum	3391	58.3
angiosperm	AJ309824.2	Zea mays	3385	58.7
