# Supplementary material for: The Expansion Segments of 28S Ribosomal RNA Extensively Match Human Messenger RNAs
Source: Front Genet. 2018 Mar 7;9:66. doi: 10.3389/fgene.2018.00066 (PMC5850279; doi:10.3389/fgene.2018.00066)
Supplement: Supplementary file 2 [file Table2.PDF]

Table S2 Boundaries of the expansion segments of human rRNAs

Boundaries of the expansion segments of human 28S rRNA

The sequence used has 5035 nt (Entrez U13369).The boundaries are adapted with minor modifications from Fig. S8 in Chandramouli et al., 2008, also consulting Table 1 in Wakeman et al., 1989. The five large ESL are labeled as V2a (for ESL7), V2b (ESL9), V5 (ESL15), V8 (ESL27) and V11 (ESL39) in Wakeman et al., 1989.

Segment	Start in sequence	End in sequence
ESL5	114	156
ESL7	465	1265
ESL9	1384	1487
ESL10	1682	1711
ESL12	1793	1829
ESL15	2075	2256
ESL19	2439	2494
ESL20	2538	2575
ESL24	2685	2705
ESL26	2751	2764
ESL27	2875	3586
ESL30	3955	4013
ESL31	4057	4124
ESL39	4698	4905
ESL41	4983	4996

Boundaries of the expansion segments of human 18S rRNA

The sequence used has 1870 nt (Entrez K03432). The boundaries are adapted with minor modifications from Figure S7 in Chandramouli et al.(2008) .

Segment	Start in sequence	End in sequence
ESS1	53	73
ESS2	116	130
ESS3	208	291
ESS4	512	583
ESS6	737	911
ESS7	1097	1114
ESS8	1272	1305
ESS9	1398	1403
ESS10	1533	1551
ESS11	1563	1581
ESS12	1726	1789

References for Table S2

Chandramouli, P., Topf, M., Menetret, J.F., Eswar, N., Cannone, J.J., Gutell, R.R., Sali, A., and Akey, C.W. (2008). Structure of the mammalian 80S ribosome at 8.7 A resolution. *Structure* 16**,** 535-548.

Wakeman, J.A., and Maden, B.E. (1989). 28 S ribosomal RNA in vertebrates. Locations of large-scale features revealed by electron microscopy in relation to other features of the sequences. *Biochem J* 258**,** 49-56.
