# Supplementary material for: The Expansion Segments of 28S Ribosomal RNA Extensively Match Human Messenger RNAs
Source: Front Genet. 2018 Mar 7;9:66. doi: 10.3389/fgene.2018.00066 (PMC5850279; doi:10.3389/fgene.2018.00066)
Supplement: Supplementary file 3 [file Table3.PDF]

Table S3 7-nucleotide antisense matches of human mRNAs to segments of human 28S and 18S rRNAs

The examined human mRNAs (18810) represent 17392 protein-coding genes, with multiple cases of non-coding sector variants. The sequences were retrieved in August of 2015 from Ensembl database (http://www.ensembl.org).

RNA and segment	Segment label used	#Nt in segment	mRNAs matched at 7 nt	% mRNAs matched	GC% match	GC% mRNA	GC% in segment
hsa28S-ES5	ESL5	43	15341	88.21	85.34	59.49	79.07
hsa28S-ES7	ESL7	801	17081	98.21	84.28	59.07	83.77
hsa28S-ES9	ESL9	104	16897	97.15	82.15	58.84	79.808
hsa28S-ES10	ESL10	30	15102	86.83	81.32	58.56	76.666
hsa28S-ES12	ESL12	37	14374	82.65	80.18	58.72	78.379
hsa28S-ES15	ESL15	182	17039	97.97	84.43	59.23	84.066
hsa28S-ES19	ESL19	56	16535	95.07	75.42	57.63	73.214
hsa28S-ES20	ESL20	38	14937	85.88	76.93	57.93	71.052
hsa28S-ES24	ESL24	21	13714	78.85	83.9	59.58	80.952
hsa28S-ES26	ESL26	14	9359	53.81	72.02	57.52	64.285
hsa28S-ES27	ESL27	712	17099	98.32	86.82	59.64	86.517
hsa28S-ES30	ESL30	59	16366	94.1	91.11	60.32	91.526
hsa28S-ES31	ESL31	68	16187	93.07	87.63	60.06	83.824
hsa28S-ES39	ESL39	208	17064	98.11	84.81	59.15	82.692
hsa28S-ES41	ESL41	14	10488	60.3	73.62	57.13	71.429
hsa28S-CS5	CSL5	113	17034	97.94	52.58	52.7	52.212
hsa28S-CS7	CSL7	308	17086	98.24	64.64	54.85	61.364
hsa28S-CS9	CSL9	118	17080	98.21	71.17	56.37	66.949
hsa28S-CS10	CSL10	194	17084	98.23	60.78	53.73	57.732
hsa28S-CS12	CSL12	81	16906	97.21	44.26	50.45	39.506
hsa28S-CS15	CSL15	245	17070	98.15	61.48	54.29	59.184
hsa28S-CS19	CSL19	182	17061	98.1	63.83	55.07	59.341
hsa28S-CS20	CSL20	43	16529	95.04	64.53	54.47	62.791
hsa28S-CS24	CSL24	109	17059	98.09	64.43	55.2	66.055
hsa28S-CS26	CSL26	45	16383	94.2	65.49	55.11	64.444
hsa28S-CS27	CSL27	110	17029	97.91	53.66	52.04	53.636
hsa28S-CS30	CSL30	368	17116	98.41	52.91	52.54	51.359
hsa28S-CS31	CSL31	43	16559	95.21	49.54	51.9	48.837
hsa28S-CS39	CSL39	573	17083	98.22	55.44	52.77	52.705
hsa28S-CS41	CSL41	77	16986	97.67	63.2	54.09	58.442
hsa28S-CSend	CSLend	39	16489	94.81	52.48	52.15	48.718
hsa18S-ES1	ESS1	21	13582	78.09	69.63	55.96	61.904
hsa18S-ES2	ESS2	15	13935	80.12	62.27	55.06	60
hsa18S-ES3	ESS3	84	16939	97.4	76.46	57.35	65.476
hsa18S-ES4	ESS4	72	16885	97.08	44.1	50.35	40.278
hsa18S-ES6	ESS6	175	17061	98.1	53.34	53.14	52.571
hsa18S-ES7	ESS7	18	9449	54.33	43.9	49.48	55.555
hsa18S-ES8	ESS8	34	15037	86.46	38.75	48.98	38.235
hsa18S-ES10	ESS10	19	10732	61.71	62.78	54.18	57.895
hsa18S-ES11	ESS11	19	12888	74.1	55.4	51.7	63.158
hsa18S-ES12	ESS12	64	16733	96.21	80.13	58.26	79.365
hsa18S-CS1	CSS1	52	15557	89.45	38.47	49.17	42.308
hsa18S-CS2	CSS2	42	15803	90.86	38.86	48.81	42.857
hsa18S-CS3	CSS3	77	16842	96.84	66.73	55.3	57.143
hsa18S-CS4	CSS4	220	17095	98.29	67.54	55.34	62.273
hsa18S-CS6	CSS6	153	17031	97.92	66.77	55.89	61.438
hsa18S-CS7	CSS7	185	17082	98.22	59.78	53.69	54.595
hsa18S-CS8	CSS8	157	17058	98.08	60.16	53.76	57.962
hsa18S-CS9	CSS9	92	16986	97.67	53.21	51.83	51.087
hsa18S-CS10	CSS10	129	17054	98.06	63.66	54.68	61.24
hsa18S-CS11	CSS11	11	6613	38.02	91.7	61.34	90.909
hsa18S-CS12	CSS12	144	17060	98.09	55.32	52.59	50.694
hsa18S-CSend	CSSend	81	16966	97.55	47.02	50.95	46.914

Lists of mRNA sector transcripts are being continuously expanded and updated for most genomes, and human genome lists could be among the most dynamic. The number of first-listed transcripts for complete mRNAs (i.e. mRNAs having all three sectors in same-numbered gene and transcript sequence record, and also bearing the lowest transcript number) in Ensembl database in July of 2017 was 20614. The additional 1804 complete mRNAs represent an increase of 9.6% over the 2015 collection that was characterized in this work. *Maximal* differences from common mean in the numbers of matches per mRNA and ES nucleotide between the 2017 and the 2015 collection are 0.945 (5'utr), 3.47 (cds) and 2.48% (3'utr) among ESL, and 1.76 (5'utr), 3.2 (cds) and 2.46% (3'utr) among ESS. The *average* difference is below 1% with 5'utr and 3'utr, and about 3% with cds, and the increase in the total number of matches was about 6%. The 2015 mRNA collection thus appears to be sufficiently representative of the potential for rRNA matching.
