# Supplementary material for: The Expansion Segments of 28S Ribosomal RNA Extensively Match Human Messenger RNAs
Source: Front Genet. 2018 Mar 7;9:66. doi: 10.3389/fgene.2018.00066 (PMC5850279; doi:10.3389/fgene.2018.00066)
Supplement: Supplementary file 4 [file Table4.PDF]

Table S4 The size and GC content of the large LSU (25-28S) rRNA segments CSL15, ESL15 and CSL19 across eukaryote species.

RNA group	CSL15	ESL15	CSL19	GC% in CS15L	GC% in ES15L	GC% in CS19L
hominid [4]	244	164.8	181.8	59.12	81.86	59.70
rodent [2]	244	131	182	59.22	84.43	59.34
bovid [2]	245	87	181.5	50.61	65.08	53.45
bird [1]	245	22	182	60.41	81.82	62.64
amphibian [1]	245	24	182	59.18	75.00	61.54
fish [3]	241.7	27.67	181.3	59.31	65.43	60.85
chordate [2]	239	33.5	183.5	59.00	67.12	58.58
mollusk [1]	245	24	181	54.69	66.67	58.01
insect-1 [4]	283	47.25	169.8	51.74	62.97	54.26
insect-2 [3]	260.7	48.67	184.3	46.62	18.51	44.99
sponge [3]	243.3	26	160.3	53.84	55.10	51.80
nematode [1]	242	22	181	51.24	59.09	50.83
fungal-1 [4]	240.5	16.5	178.5	50.73	40.65	50.82
fungal-2 [3]	241.7	40.33	152.3	54.21	64.36	53.82
lower eukarya [2]	235.5	41	191.5	52.29	48.53	49.67
angiosperm [4]	242	14	176.8	56.40	66.07	58.67
mean	*246.1*	*48.1*	*178.1*	*54.91*	*62.67*	*55.56*
sd	*11.14*	*43.13*	*9.649*	*4.162*	*16.59*	*5.063*
se	*2.786*	*10.78*	*2.412*	*1.04*	*4.148*	*1.266*
min	*235.5*	*14*	*152.3*	*46.62*	*18.51*	*44.99*
max	*283*	*164.8*	*191.5*	*60.41*	*84.43*	*62.64*
n	*16*	*16*	*16*	*16*	*16*	*16*
% cv	*4.53*	*89.67*	*5.42*	*7.58*	*26.47*	*9.11*
