# Supplementary material for: The Expansion Segments of 28S Ribosomal RNA Extensively Match Human Messenger RNAs
Source: Front Genet. 2018 Mar 7;9:66. doi: 10.3389/fgene.2018.00066 (PMC5850279; doi:10.3389/fgene.2018.00066)
Supplement: Supplementary file 5 [file Table5.PDF]

Table S5 Matches of 16-20-nucleotides with human mRNAs in segments of human 28S and 18S rRNAs

Segment	16-19 nt	20 nt
ESL5	1	0
ESL7	5664	329
ESL9	14	0
ESL10	1	0
ESL15	6464	516
ESL27	1859	18
ESL30	2	0
ESL31	6	0
ESL39	34	0
total	**14045**	**863**
CSL9	20	0
CSL10	6	0
CSL12	2	0
CSL15	1	0
CSL19	4	0
CSL20	1	0
CSL24	9	0
CSL26	1	0
CSL30	4	0
CSL39	21	0
CSLend	1	0
total	**70**	**0**
ESS4	15	0
ESS6	2	0
ESS12	3	0
total	**20**	**0**
CSS4	4	0
CSS6	19	0
CSS7	11	0
CSS8	3	0
CSS9	11	**0**
CSS10	1	0
CSS12	4	0
CSSend	1	0
total	**54**	**0**

No 16-20-nt matches found in:
ESL12, 19, 20, 24, 26 and 41
CSL5, 7, 27, 31 and 41
ESS1, 2, 3, 7, 8, 10 and 11
CSS1, 2, 3 and 11
