# Supplementary material for: The Expansion Segments of 28S Ribosomal RNA Extensively Match Human Messenger RNAs
Source: Front Genet. 2018 Mar 7;9:66. doi: 10.3389/fgene.2018.00066 (PMC5850279; doi:10.3389/fgene.2018.00066)
Supplement: Supplementary file 6 [file Table6.PDF]

Table S6 The *scramble* function for scrambling oligo- or polynucleotide sequences

```
Public Function scramble(Target As Variant)
' by Mr. Hui  from https://Chandoo.org
' Call by e.g. placing the sequence to scramble into cell A1
' and typing "=scramble(A1)" in cell A2 and pressing Enter.
' The resulting shuffle will display in cell A2.
' Further scrambles can be obtained by duplicating the first one
' into the same cell of succeeding rows.
' The result depends on randomization linked to the timer.
' For multiple scrambles, it is important to assure that there are no
' copying and pasting mistakes; i.e., the results ought to have
' the same length as the original sequence, and the same GC content.


On Error Resume Next
Dim CL As New Collection
Application.Volatile

Do Until CL.Count = Len(Target)
r = Int(1 + Rnd * Len(Target))
CL.Add r, CStr(r)
Loop

For i = 1 To CL.Count
scramble = scramble & Mid(Target, CL(i), 1)
Next

End Function
```
